# Biallelic Cys141Tyr variant of *SEL1L* is associated with neurodevelopmental disorders, agammaglobulinemia, and premature death

**DOI:** 10.1172/JCI170882

**Published:** 2024-01-16

**Authors:** Denisa Weis, Liangguang L. Lin, Huilun H. Wang, Zexin Jason Li, Katarina Kusikova, Peter Ciznar, Hermann M. Wolf, Alexander Leiss-Piller, Zhihong Wang, Xiaoqiong Wei, Serge Weis, Katarina Skalicka, Gabriela Hrckova, Lubos Danisovic, Andrea Soltysova, Tingxuan T. Yang, René Günther Feichtinger, Johannes A. Mayr, Ling Qi

**Affiliations:** 1 Department of Medical Genetics, Kepler University Hospital, School of Medicine, Johannes Kepler University, Linz, Austria.; 2 Department of Pediatrics, Faculty of Medicine, Comenius University Bratislava and National Institute of Children’s Diseases, Bratislava, Slovakia.; 3 Department of Molecular Physiology and Biological Physics, University of Virginia, Charlottesville, Virginia, USA.; 4 Department of Molecular & Integrative Physiology and; 5 Department of Biological Chemistry, University of Michigan Medical School, Ann Arbor, Michigan, USA.; 6 Department of Pediatric Neurology, Faculty of Medicine, Comenius University Bratislava and National Institute of Children’s Diseases, Bratislava, Slovakia.; 7 Immunology Outpatient Clinic, Vienna, Austria.; 8 Sigmund Freud Private University–Medical School, Vienna, Austria.; 9 Division of Neuropathology, Neuromed Campus, Department of Pathology and Molecular Pathology, Kepler University Hospital, Johannes Kepler University, Linz, Austria.; 10 Institute of Medical Biology, Genetics and Clinical Genetics, Faculty of Medicine, and; 11 Faculty of Natural Sciences, Department of Molecular Biology, Comenius University, Bratislava, Slovakia.; 12 Institute for Clinical and Translational Research, Biomedical Research Centre, Slovak Academy of Sciences, Bratislava, Slovakia.; 13 University Children’s Hospital, Salzburger Landeskliniken Universitätsklinikum (SALK) and Paracelsus Medical University (PMU), Salzburg, Austria.

**Keywords:** Cell Biology, Adaptive immunity, Genetic diseases, Protein misfolding

## Abstract

Suppressor of lin-12-like–HMG-CoA reductase degradation 1 (SEL1L-HRD1) ER-associated degradation (ERAD) plays a critical role in many physiological processes in mice, including immunity, water homeostasis, and energy metabolism; however, its relevance and importance in humans remain unclear, as no disease variant has been identified. Here, we report a biallelic *SEL1L* variant (p. Cys141Tyr) in 5 patients from a consanguineous Slovakian family. These patients presented with not only ERAD-associated neurodevelopmental disorders with onset in infancy (ENDI) syndromes, but infantile-onset agammaglobulinemia with no mature B cells, resulting in frequent infections and early death. This variant disrupted the formation of a disulfide bond in the luminal fibronectin II domain of SEL1L, largely abolishing the function of the SEL1L-HRD1 ERAD complex in part via proteasomal-mediated self destruction by HRD1. This study reports a disease entity termed ENDI-agammaglobulinemia (ENDI-A) syndrome and establishes an inverse correlation between SEL1L-HRD1 ERAD functionality and disease severity in humans.

## Abstract 2

A biallelic SEL1L C141Y variant causes premature death in 5 patients with early-onset neurodevelopmental disorders and agammaglobulinemia (ENDI-A) due to severe SEL1L-HRD1 ER-associated degradation dysfunction.

## Graphical abstract

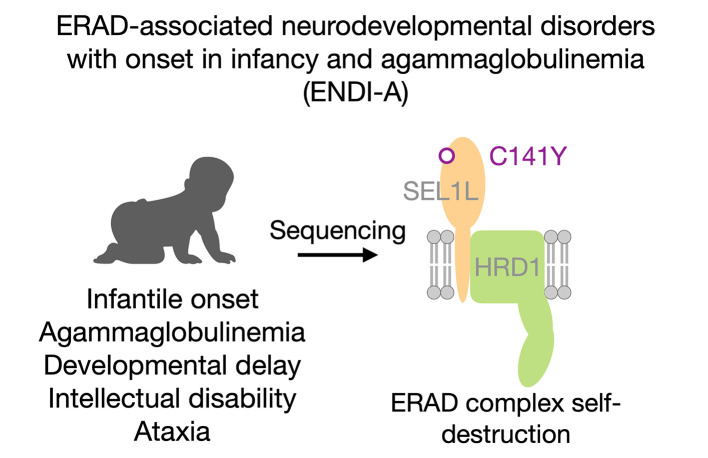

## Introduction

ER-associated degradation (ERAD) is the key cellular quality-control mechanism underlying the clearance of misfolded proteins from the ER, thereby generating a conducive environment for protein folding, maturation, and maintaining ER homeostasis. The suppressor of lin-12-like–HMG-CoA reductase degradation 1 (SEL1L-HRD1) complex, together with lectin osterosarcoma amplified 9 (OS9), ER lectin 1 (ERLEC1, also known as XTP3B), and degradation in ER (DERLIN) proteins, represents one of the most conserved branches of ERAD (1–6). In vivo, global or acute deletion of *Sel1L* or *Hrd1* in germline and adult mice causes embryonic or premature lethality, respectively (7–10). Subsequent studies using cell type–specific KO mouse models, including those from our group, have established the vital importance of SEL1L-HRD1 ERAD in different cell types, including hematopoietic stem cells, various immune cells, pancreatic β cells, podocytes, hepatocytes, and adipocytes, in many physiological processes (4–6, 8, 11–31). However, its relevance and importance in humans remain unexplored.

As the folding capacity within the ER varies greatly among different cell types, it has been hypothesized that cells may exhibit differential dependency on SEL1L-HRD1 ERAD (4–6). However, to date, the molecular evidence for this model remains circumstantial and a challenging question for the field. Intriguingly, we and others recently reported that SEL1L-HRD1 ERAD is indispensable for B cell development by targeting pre–B cell receptor (pre-BCR) for proteasomal degradation (11, 13). However, in mature B cells, which secrete large amounts of immunoglobulin (Ig) proteins, SEL1L-HRD1 ERAD function seems to be dispensable (11). In the absence of SEL1L, B cell development is blocked at the large pre–B cell stage due to the accumulation of pre-BCR at the cell surface (11, 13). In contrast, SEL1L deficiency in developing T cells attenuated, but failed to block, the development of αβ T cells while having no effect on that of γδ T cells (15). Hence, cell type–specific dependency of SEL1L-HRD1 ERAD in mammals remains an open, but exciting, question.

In the accompanying paper, we reported 3 hypomorphic biallelic *SEL1L* and *HRD1* variants causing a group of inherited disorders in 6 patients with ERAD-associated neurodevelopmental disorders with onset in infancy (ENDI) (32). Here, we report another *SEL1L* variant, p.Cys141Tyr (*SEL1L^C141Y^*), in 5 patients from a large family, with similar ENDI. However, unlike the other ENDI patients who are in their teens and 20s, these 5 patients exhibited B cell depletion and agammaglobulinemia and died at very early ages as a result of frequent infections. Mechanistic studies showed that this variant caused the most severe SEL1L-HRD1 ERAD dysfunction among all 4 variants by causing disulfide bond–mediated aggregation and HRD1-mediated degradation of SEL1L. These studies together demonstrate that functionality of SEL1L-HRD1 ERAD is inversely correlated with disease severity in humans.

## Results

### Identification of a biallelic SEL1L^C141Y^ variant in humans.

**Figure 1:**
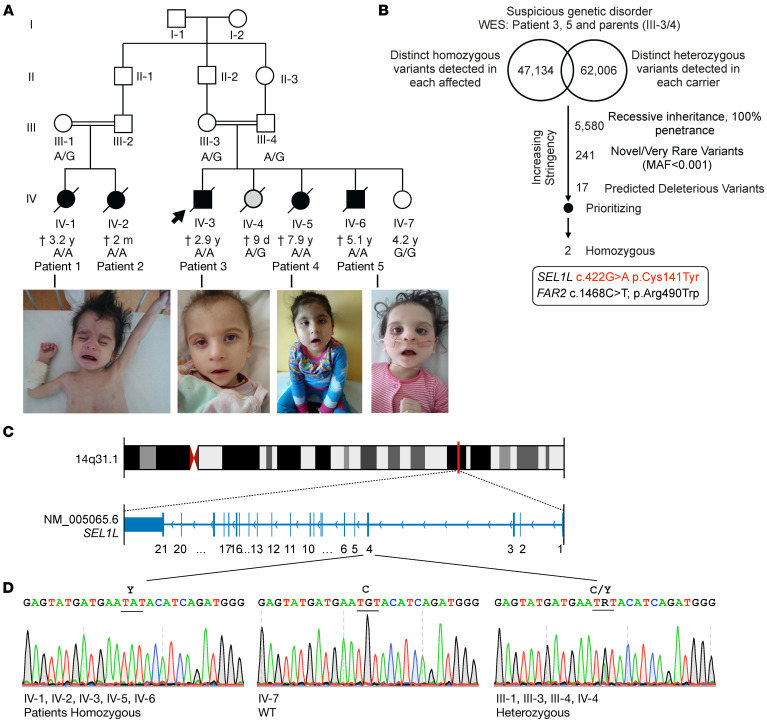
Identification of *SEL1L^C141Y^* variant in humans using whole-exome sequencing. (**A**) Family pedigrees for the kindreds from Slovakia (2 consanguineous pairs) for *SEL1L* p.C141Y, showing autosomal recessive inheritance. Arrows point to probands. Black shapes indicate affected patients and gray shapes show a newborn died from holoprosencephaly. The age indicated is as of 2022 or at time of death (cross). Photos of the patients are shown below. Photo of patient 1 was published in the book *Pediatrics* (51) to show marasmus and is republished here with permission. (**B**) Genetic analysis pipeline of whole-exome sequencing (WES) data for patients 3 and 5 (IV-3/6) and their parents, III-3/4. (**C** and **D**) Exonic and chromosomal locations of *SEL1L* variant (**C**), with Sanger sequencing in patients and other family members (**D**). R, heterozygosity; C, cysteine; Y, tyrosine.

Five siblings from 2 consanguineous families in a large Slovakian family presented with developmental delay, neurological disorders, and agammaglobulinemia in childhood (Figure 1) and were suspected of inherited genetic disorder. Array comparative genomic hybridization (aCGH) analysis performed in patients 4 and 5 did not reveal the presence of any larger deletions or amplifications within the genome (Supplemental Figure 1; supplemental material available online with this article; https://doi.org/10.1172/JCI170882DS1). Analyses of whole-exome sequencing of DNA samples from patients 3 and 5 and their parents (III-3 and III-4) did not identify any known monogenic inborn errors of neurological disorders and agammaglobulinemia. Since both patients were born to the same consanguineous parents, variants were queried from the database according to the assumption of a recessive inheritance with 100% penetrance. Annotated variants were then filtered against their allele frequency (smaller than 1% or unknown) and predicted deleteriousness (Figure 1B). Two variants were identified in individual patients, *SEL1L* p.Cys141Tyr (NM_005065.6: exon 4: c.422G>A) and fatty acyl-CoA reductase 2 (*FAR2*) p.Arg490Trp (NM_001271783.2: exon 12: c.1468C>T) (Figure 1B and Supplemental Table 1). As Sanger sequencing confirmed the segregation of the *FAR2* variant with symptoms in patient 2 (Supplemental Figure 2) and as loss of FAR2 function is not linked to neurological disorder or agammaglobulinemia (33), we excluded it from being causal for these patients. Moreover, Sanger sequencing further confirmed the biallelic *SEL1L^C141Y^* variant in all 5 patients, but not in parents or unaffected siblings (Figure 1, C and D). Hence, on the basis of the known function of SEL1L protein, and after applying stringent filters of the exome sequencing data (Supplemental Table 1) according to the American College of Medical Genetics and Genomics (ACMG) and the Association for Molecular Pathology (AMP) 2015 guidelines for clinical interpretation of genetic variants (34), we determined that *SEL1L^C141Y^* is a potential candidate.

### SEL1L^C141Y^ variant in patients with ENDI-agammaglobulinemia.

The clinical presentation was uniform among the 5 patients with ENDI-agammaglobulinemia (ENDI-A). All patients started to have problems with food intake soon after birth, as they repeatedly vomited after eating (Table 1 and Supplemental Table 2). They were unable to gain weight, had pale skin color, gradually developed cachexia, and had similar facial dysmorphisms, including triangle faces, big ears, etc. (Figure 1A). All patients showed severe axial hypotonia and general developmental delay with short stature and microcephaly (Table 1). Three patients (patients 1, 3, and 5) could not sit up, hold their heads, or raise their heads while supporting themselves on their elbows. Only patient 4 was able to sit without support at the age of 4.5 years. All patients exhibited intellectual disability and were unable to speak words and sentences (Table 1 and Supplemental Table 2). Of note, the IV-4 individual, carrying 1 allele *SEL1L^C141Y^*, was born with multiple malformations (alobar holoprosencephaly, “frog” eyes, anus malformation, congenital heart defects, etc.) and died from cardiorespiratory failure at 9 days of age (Figure 1A). Hence, these patients exhibited typical ENDI symptoms as described in the accompanying paper (Table 1) (32).

However, unlike the other ENDI patients, these patients were frequently admitted to the hospital due to recurrent severe lower respiratory infection almost every month, starting from otitis media, sinusitis (bilateral maxillary and ethmoidal), to bronchitis and pneumonia, as diagnosed by doctors. During pneumonia, children developed respiratory insufficiency often caused by food aspiration. As a result, they were hospitalized nearly monthly and treated with antibiotics and sufficient oxygen supply and were regularly supplemented with Igs. In patients 3 and 4, hearing impairment was diagnosed at 1 year of age, which was likely secondary to multiple otitis media.

Immunological tests were performed with peripheral blood from patients 3, 4, and 5, which revealed no detectable mature CD19^+^ memory B cells in the circulation or circulating Igs at the age of 12 months and beyond (Table 2). Consequently, IgG was given as a replacement therapy every 4 weeks in these patients. Indeed, after replacement therapy was initiated, a substantial improvement in acute infections was noted. However, chronic respiratory symptoms, phlegm, and cough in some forms continued to recur. The early treatment strategy by IgG replacement therapy and antiinfectious prophylaxis very likely postponed infectious complications in patients 3, 4, and 5. However, all 3 were observed to have a sudden dramatic deterioration of the clinical condition due to sepsis with multiorgan failure.

A gut biopsy of patient 3 showed subtotal villous atrophy of the duodenum as Marsh IIIb enteropathy with no CD20^+^ B cells and moderate to high amounts of CD3^+^, CD4^+^, and CD8^+^ intraepithelial cells compared with noncarriers (Supplemental Figure 3A and Supplemental Table 3). Although the absolute numbers of CD4^+^ helper and CD8^+^ cytotoxic T cells were largely in the normal ranges, the ratio of CD4^+^ to CD8^+^ T cells was reduced (Table 2), pointing to an impaired T cell development with defective SEL1L-HRD1 ERAD. Moreover, patient 4 had a COVID-19 infection with respiratory failure at the age of 7.7 years. After the patient was administered with therapeutic anti–SARS-Cov-2 monoclonal antibodies, the viral load was effectively decreased. However, the course of treatment was prolonged and differed from that of healthy children or children with X-linked agammaglobulinemia (XLA). Indeed, no specific cellular response measured by COVID antigen–specific memory T cells was detected in peripheral blood (Supplemental Figure 3B), indicative of defects in COVID antigen–induced T cell activation. Hence, these patients lacked mature B cells and exhibited agammaglobulinemia, with impaired T cell development and/or function.

### Sequence and structural analyses of SEL1L^C141Y^ variant.

**Figure 2:**
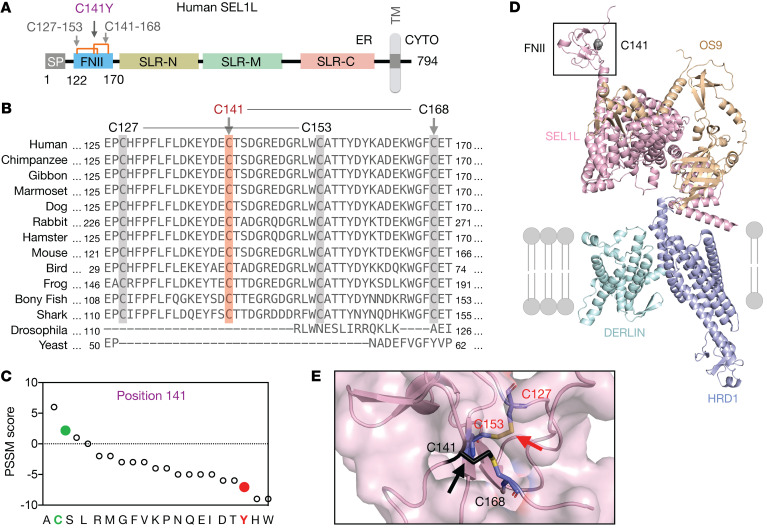
Sequence and structural analyses of *SEL1L^C141Y^* variant. (**A**) Schematic diagram of human SEL1L. SP, signal peptide; SLR-N/M/C, Sel1-like repeats at N-, middle-, and C-terminal; TM, transmembrane; CYTO, cytosol. Orange lines, 2 disulfide bonds (Cys127-Cys153, Cys141-Cys168). (**B**) The aa sequence alignment of SEL1L showing the conservation of SEL1L C141 residue (highlighted in orange) and neighboring cysteine residues (highlighted in gray) across species. (**C**) PSSM scores for position 141, with WT in green and variant in red. (**D** and **E**) Structural prediction of human SEL1L/OS9/HRD1/DERLIN ERAD complex using AlphaFold2 with close-up view of C141 residue and disulfide bonds (black arrows) (**E**).

This variant affects a conserved residue in the luminal N-terminal fibronectin type II (FNII) domain of SEL1L, a domain with unknown function (Figure 2A). Interestingly, unlike other domains of SEL1L, the FNII domain is not conserved in invertebrates (Figure 2B). Position-specific scoring matrix (PSSM) analysis (35) showed that Cys at this position was evolutionarily selected and that the Cys-to-Tyr mutation may be detrimental to SEL1L function (Figure 2C). Structural modeling of the human SEL1L-HRD1-OS9-DERLIN1 protein complex (SEL1L, 107–723 aa; HRD1, 1–334 aa; OS9, 33–655 aa; DERLIN1, 1–213 aa) using the AI-based AlphaFold2 prediction network (36) showed 2 short antiparallel β sheets connected by 2 long random coils at the FNII domain with 2 disulfide bridges in close, quasiorthogonal juxtaposition (Figure 2, D and E): Cys141-Cys168 and Cys127-Cys153.

### SEL1L^C141Y^ variant causes ERAD complex instability and dysfunction.

**Figure 3:**
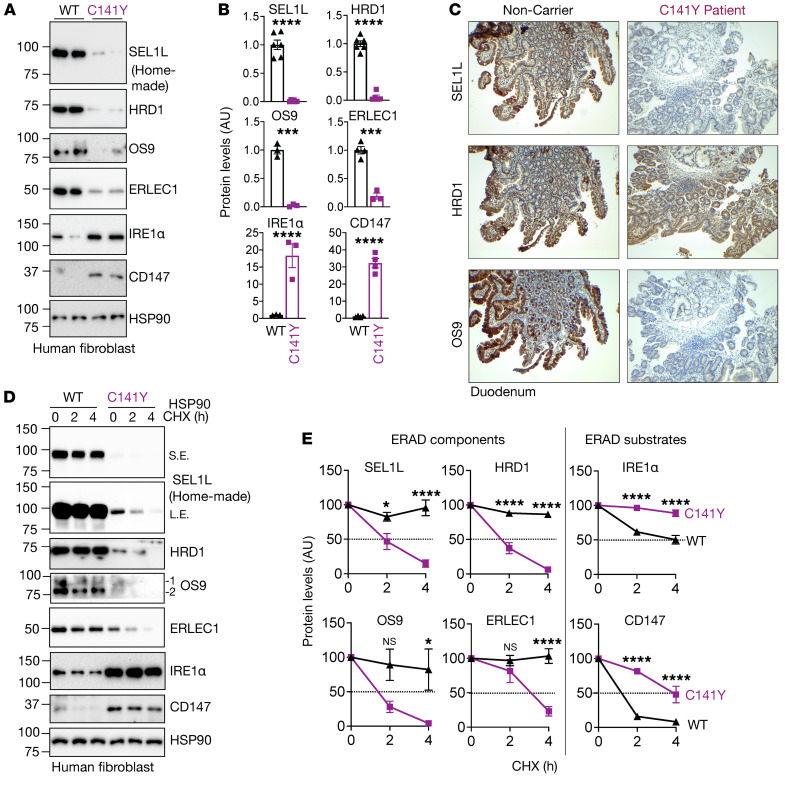
*SEL1L^C141Y^* variant abolishes ERAD complex and function. (**A** and **B**) Western blot analysis of ERAD proteins and endogenous ERAD substrates in WT and *SEL1L^C141Y^* patient fibroblasts with quantitation shown (**B**) (*n* = 3–6 per group). (**C**) Immunohistochemical staining of SEL1L (top), HRD1 (middle), and OS9 (bottom) in duodenal biopsies from patient 3 and noncarrier control. Original magnification, ×40. (**D** and **E**) Cycloheximide (CHX) chase analysis of ERAD proteins and endogenous ERAD substrates in WT and *SEL1L^C141Y^* patient fibroblasts with quantitation shown (**E**) (*n* = 3–6 per group). OS9 1 indicates isoform OS-9.1; OS9 2 indicates isoform OS-9.2. Both bands were quantitated together. *n*, individual cell samples. Data are represented as means ± SEM. *P < 0.05; ***P < 0.001; ****P < 0.0001, 2-tailed Student’s *t* test (**B**); 2-way ANOVA followed by Tukey’s multiple-comparisons test (**E**).

We next tested to determine whether and how *SEL1L^C141Y^* affects ERAD function using skin fibroblasts derived from patients and those from noncarrier individuals as WT controls. Strikingly, SEL1L protein levels were significantly reduced and largely undetectable in the patient fibroblasts compared with in WT cells (Figure 3, A and B). In keeping with our previous findings that SEL1L is required for HRD1 protein stability (37), HRD1 protein levels were also significantly reduced, by over 90%, in patient cells (Figure 3, A and B). The reduction of SEL1L and HRD1 protein levels was confirmed using immunofluorescent staining in patient skin fibroblasts (Supplemental Figure 4, A and B) as well as immunohistochemical staining of the duodenal biopsies from the patients (Figure 3C). This reduction in SEL1L-HRD1 protein levels was not due to gene transcription, as their mRNA levels were unchanged compared with those in healthy cells (Supplemental Figure 4C). SEL1L and HRD1 proteins became unstable in *SEL1L^C141Y^* patient cells treated with a translation inhibitor, cycloheximide (Figure 3, D and E), while 2 known ERAD substrates, inositol-requiring enzyme 1α (IRE1α) (37) and CD147 (38), were accumulated and stabilized in *SEL1L^C141Y^* patient cells (Figure 3, A, B, D, and E). Much to our surprise, 2 lectins that help recruit substrates to the SEL1L-HRD1 complex, OS9 and ERLEC1, were significantly decreased and destabilized in *SEL1L^C141Y^* patient cells (Figure 3, A–E), uncoupled from their gene transcription (Supplemental Figure 4C). Furthermore, a model ERAD substrate proarginine vasopressin (proAVP) mutant, Gly57Ser (proAVP G57S) (24), formed significantly more high–molecular weight (HMW) aggregates in *SEL1L^C141Y^* knockin (KI) HEK293T cells compared with WT cells, to levels similar to those in ERAD-KO HEK293T cells (Supplemental Figure 5, A and B). ER staining showed an increase in ER volume in *SEL1L^C141Y^* KI HEK293T cells (Supplemental Figure 5C).

### SEL1L^C141Y^ causes the most severe ERAD dysfunction among all 4 variants.

**Figure 4:**
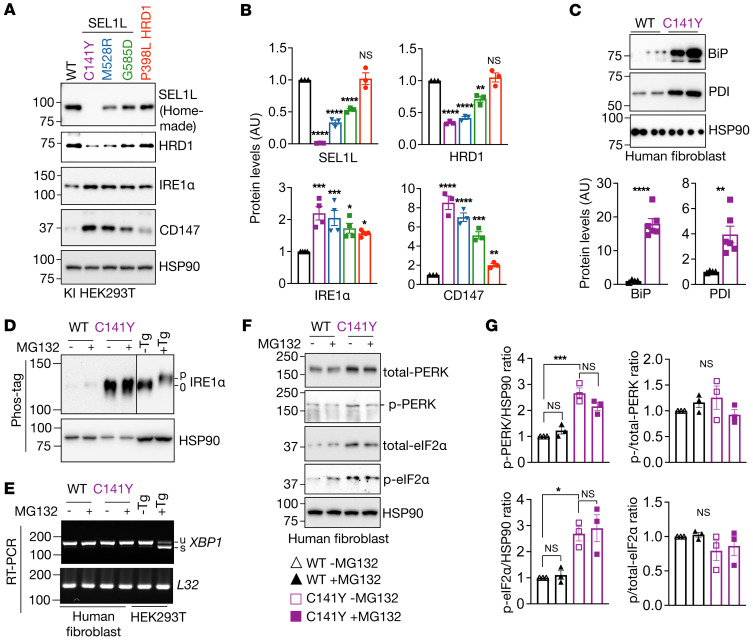
*SEL1L^C141Y^* causes severe ERAD dysfunction, but not an overt UPR. (**A** and **B**) Western blot analysis of SEL1L, HRD1, and endogenous ERAD substrates in various KI HEK293T cells expressing different variants, with quantitation shown (**B**) (*n* = 3–4 per group). (**C**) Western blot analysis of ER chaperones in WT and *SEL1L^C141Y^* patient fibroblasts with quantitation shown below (*n* = 4–6 per group). (**D**) Western blot analysis of IRE1α phosphorylation using Phos-tag gel in WT and *SEL1L^C141Y^* patient fibroblasts treated with and without 10 μM MG132 for 2 hours (*n* = 3 per group). Tg, thapsigargin, ER stress inducer. (**E**) Reverse transcription PCR (RT-PCR) of *XBP1* splicing levels in WT and *SEL1L^C141Y^* patient fibroblasts treated with and without 10 μM MG132 for 2 hours. Two independent repeats. u, unsplicing; s, splicing. (**F** and **G**) Western blot analysis of PERK and eIF2α phosphorylation in WT and *SEL1L^C141Y^* patient fibroblasts treated with and without 10 μM MG132 for 2 hours, with quantitation shown (**G**) (*n* = 3 per group). p, phosphorylation. *n*, individual cell samples. Data are represented as means ± SEM. *P < 0.05; **P < 0.01; ***P < 0.001; ****P < 0.0001, 2-tailed Student’s *t* test (**B**, CD147 protein level comparison between WT and P398L cells; **C**); 1-way ANOVA followed by Tukey’s post hoc test (**B**, comparison between WT and other variants; **G**).

We next compared the *SEL1L^C141Y^* variant to the other hypomorphic *SEL1L* and *HRD1* variants *(SEL1L^M528R^*, *SEL1L^G585D^*, and *HRD1^P398L^*) described in the accompanying paper by Wang et al. (32) in terms of ERAD function by generating KI HEK293T cells using the CRISPR/Cas9 system expressing individual variants (Supplemental Figure 6, A–E). Indeed, compared with cells expressing other hypomorphic variants, *SEL1L^C141Y^* cells had the lowest SEL1L and HRD1 protein levels, but the highest protein levels of the ERAD substrates CD147 and IRE1α (Figure 4, A and B). Hence, we conclude that *SEL1L^C141Y^*-expressing cells exhibit the most severe ERAD dysfunction among all the hypomorphic variants.

### Lack of an overt unfolded protein response in SEL1L^C141Y^ cells.

We next asked whether ERAD dysfunction in *SEL1L^C141Y^* cells induces an overt unfolded protein response (UPR). UPR was measured using standard protocols as previously described (39). ER chaperones Ig heavy chain–binding protein (BiP) and protein disulfide isomerase (PDI) were significantly elevated (Figure 4C). While IRE1α protein levels were elevated in *SEL1L^C141Y^* cells, IRE1α was not phosphorylated based on the phos-tag system (39, 40) (Figure 4D). Consistently, X-box–binding protein 1 (*XBP1*) mRNA splicing was not detected in *SEL1L^C141Y^* cells (Figure 4E). Although the absolute levels of phosphorylation of protein kinase R-like ER kinase (PERK) and eukaryotic initiation factor 2α (eIF2α) were elevated in patient fibroblasts when normalized to the loading control HSP90, there was no difference in the percentage of phosphorylated PERK and eIF2α upon normalization to total PERK and eIF2α proteins (Figure 4, F and G). Changes in these markers were not affected by the treatment of MG132 (Figure 4, D and G). Hence, we conclude that *SEL1L^C141Y^* causes severe ERAD dysfunction, but is not associated with an overt UPR. This scenario likely resulted from the upregulation of ER chaperones and the expansion of the ER volume.

### The disulfide bonds in the FNII domain are required for ERAD complex stability and function.

**Figure 5:**
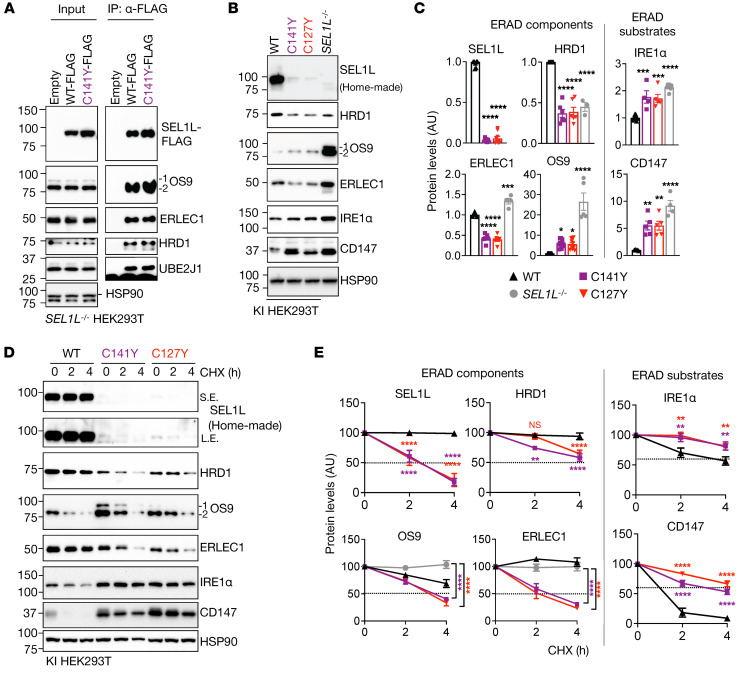
Two disulfide bond pairs in the FNII domain of SEL1L are indispensable for ERAD complex stability and function. (**A**) Immunoprecipitation of FLAG-agarose in *SEL1L^–/–^* HEK293T cells transfected with indicated SEL1L-FLAG constructs to examine their interactions with other ERAD components (*n* = 2 per group). (**B** and **C**) Western blot analysis of ERAD proteins and endogenous ERAD substrates in WT, the *SEL1L* variants KI or *SEL1L^–/–^* HEK293T cells, with quantitation shown (**C**). *n* = 5–9 (WT); *n* = 5–7 (C141Y); *n* = 5–9 (C127Y); *n* = 3–7 (*SEL1L^–/–^*). (**D**) Cycloheximide chase analysis of ERAD proteins and endogenous ERAD substrates in WT and *SEL1L* variants. (**E**) Quantitation of Figure 5D and Supplemental Figure 7. *n* = 4–10 (WT); *n* = 4–6 (C141Y); *n* = 4–6 (C127Y); *n* = 5–6 (*SEL1L^–/–^*). OS9 1 indicates isoform OS-9.1; OS9 2 indicates isoform OS-9.2. Both bands were quantitated together. *n*, individual cell samples. Data are represented as mean ± SEM. *P < 0.05; **P < 0.01; ***P < 0.001; ****P < 0.0001, 1-way ANOVA followed by Tukey’s post hoc test (**C**); 2-way ANOVA followed by Tukey’s multiple-comparisons test (**E**).

We next asked mechanistically how the *SEL1L^C141Y^* variant affects ERAD complex stability. To this end, we first performed immunoprecipitation to examine the complex formation. Upon overexpression, *SEL1L^C141Y^* had no effect on the interactions between SEL1L and other ERAD components, such as OS9, ERLEC1, HRD1, and ubiquitin-conjugating E2 enzyme J1 (UBE2J1) (Figure 5A), thus excluding the possibility that the *SEL1L^C141Y^* variant interferes with the complex formation. Next, as SEL1L has 2 disulfide bond pairs, C127-C153 and C141-C168, in the FNII domain (Figure 2A), we asked whether each disulfide bond had a similar impact on ERAD complex stability. We disrupted another disulfide bond by generating *SEL1L^C127Y^* KI HEK293T cells (Supplemental Figure 6, A–E). Indeed, similarly to *SEL1L^C141Y^*, *SEL1L^C127Y^* reduced the protein levels of the ERAD complex and stabilized the known ERAD substrates (Figure 5, B and C). Importantly, the effects of both variant/mutant on protein levels of the ERAD complex and substrates were relatively milder compared with those of *SEL1L^–/–^* HEK293T cells (Figure 5, B and C), suggesting that *SEL1L^C141Y^* is not a complete loss-of-function variant, which may explain why the patients could survive for months or even years. Moreover, cycloheximide experiments showed that both variants rendered the ERAD complex unstable while increasing the stability of ERAD substrates such as IRE1α and CD147 (Figure 5, D and E). It is worth noting that, unlike in ERAD-deficient cells, where both lectins are stabilized, OS9 and ERLEC1 were unstable in both *SEL1L^C141Y^* and *SEL1L^C127Y^* cells (Supplemental Figure 7 and Figure 5E), pointing to an additional impact of free Cys in SEL1L on other ERAD components. Hence, disulfide bonds in the FNII domain of SEL1L are indispensable for ERAD complex stability and function.

### SEL1L FNII domain is dispensable for ERAD function.

**Figure 6:**
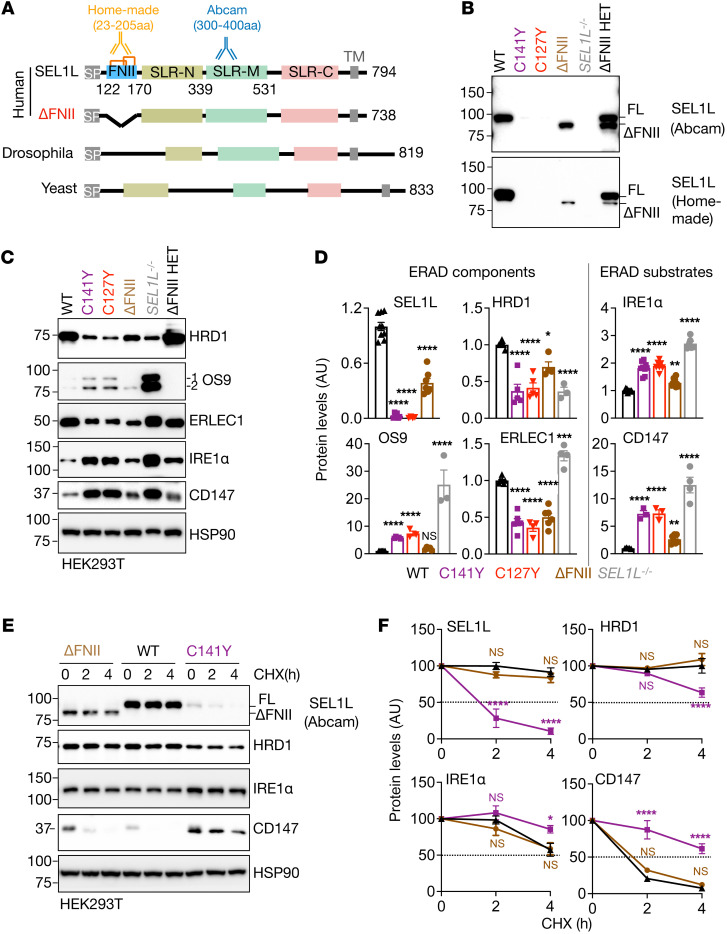
SEL1L FNII domain itself is dispensable for ERAD function. (**A**) Schematic diagrams of human full-length and FNII truncated (ΔFNII, 115–170 aa) SEL1L and its homolog Hrd3 in drosophila and yeast with the epitopes recognized by either homemade or Abcam (ab78298) SEL1L antibodies indicated. SP, signal peptide; SLR-N/M/C, Sel1-like repeats at N-, middle-, and C-terminal; TM, transmembrane; Orange lines indicate 2 disulfide bonds. (**B**–**D**) Western blot analysis of ERAD proteins and endogenous ERAD substrates in WT, *SEL1L* variants KI, ΔFNII, *SEL1L^–/–^*, and *SEL1L*^WT/ΔFNII^ (ΔFNII HET) HEK293T cells, with quantitation shown (**D**). *n* = 5–11 (WT); *n* = 3–9 (*SEL1L^C141Y^*); *n* = 3–8 (*SEL1L^C127Y^*); *n* = 4–10 (ΔFNII); *n* = 3–6 (*SEL1L^–/–^*) independent samples. OS9 1 indicates isoform OS-9.1; OS9 2 indicates isoform OS-9.2. Both bands were quantitated together. (**E** and **F**) Cycloheximide chase analysis of ERAD proteins and endogenous ERAD substrates in various KI HEK293T cells, with quantitation shown (**F**). *n* = 4–9 (WT); *n* = 3–5 (ΔFNII); *n* = 3–5 (*SEL1L^C141Y^*). Data are represented as means ± SEM. *P < 0.05; **P < 0.01; ****P < 0.0001, 1-way ANOVA followed by Tukey’s post hoc test (**D**); 2-way ANOVA followed by Tukey’s multiple-comparisons test (**F**).

We next explored the importance of the FNII domain in ERAD function. Interestingly, the FNII domain (aa 122–170) is not conserved and is absent in fly or yeast SEL1L homolog Hrd3 (Figure 6A). We generated FNII-less SEL1L HEK293T cells using CRISPR/Cas9-mediated deletion of the entire exon 4 encoding residues 115 to 170 (Supplemental Figure 8, A–C). Initial experiments using a homemade N-terminus–specific (which includes the FNII domain) antibody showed an approximately 90% reduction of SEL1L protein levels in FNII-less SEL1L KI HEK293T cells (Figure 6B). However, using a C-terminus–specific antibody (from Abcam), we noted that loss of the FNII domain caused an approximately 60% reduction of SEL1L protein levels compared with that in WT cells, which was much higher than those in cells expressing Cys variants (Figure 6, B and D). The difference between these 2 antibodies was also further confirmed in *SEL1L*^WT/ΔFNII^ cells (ΔFNII HET) (Figure 6B). These findings not only confirmed the deletion of the FNII domain in FNII-less SEL1L, but also showed that the FNII domain is important for SEL1L protein stability. By comparison, both antibodies detected very little, if any, SEL1L protein in *SEL1L^C141Y^* KI HEK293T cells (Figure 6, B and D), hence excluding the possibility that the failure to detect SEL1L protein was due to antibody recognition affected by Cys mutations.

Moreover, HRD1 protein levels in FNII-less SEL1L KI HEK293T cells were reduced by 30% compared with those in WT cells, but doubled compared with those expressing the Cys variants (Figure 6, C and D). Further examination of ERAD substrates such as IRE1α and CD147 showed mild, if any, changes in their protein levels and stability in FNII-less SEL1L KI HEK293T cells compared with WT HEK293T cells (Figure 6, C–F), pointing to largely normal ERAD function associated with FNII-less SEL1L. Hence, we conclude that the FNII domain of SEL1L is dispensable for SEL1L-HRD1 ERAD function and that the *SEL1L^C141Y^* variant affects ERAD complex stability, likely through the unpaired Cys.

### SEL1L^C141Y^ variant causes proteasome-mediated self-destruction.

**Figure 7:**
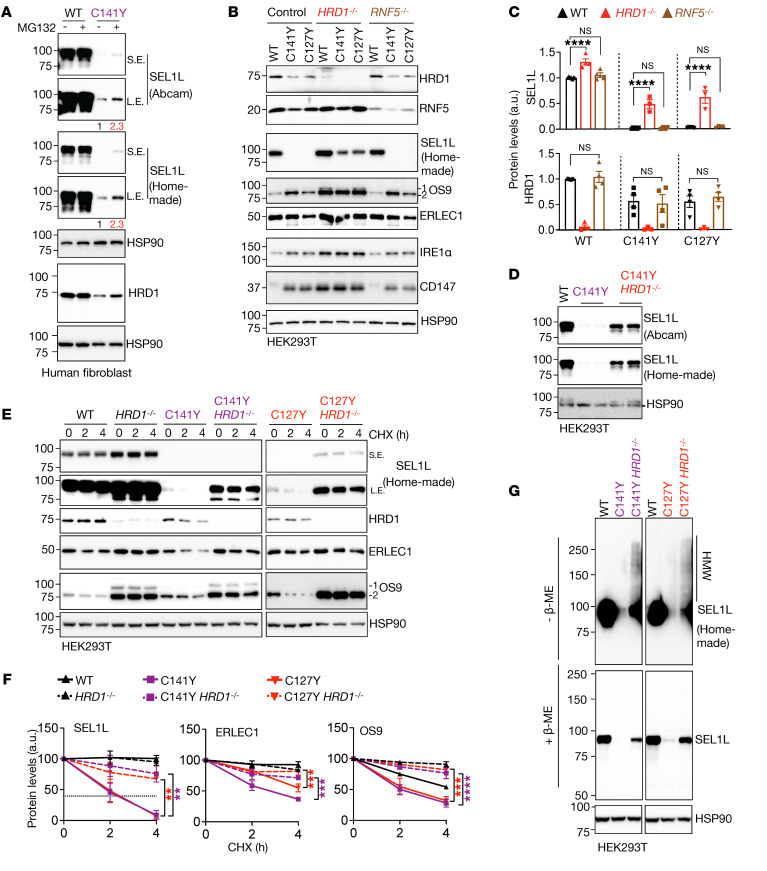
*SEL1L^C141Y^* variant causes proteasome-mediated self-destruction of SEL1L-HRD1 ERAD complex. (**A**) Western blot analysis of SEL1L and HRD1 in WT and *SEL1L^C141Y^* patient fibroblasts treated with and without 10 μM MG132 for 2 hours, with quantitation shown in Supplemental Figure 9A. *n* = 6–8 (WT); *n* = 3–4 (C141Y). (**B** and **C**) Western blot analysis of ERAD proteins and endogenous ERAD substrates in WT or KI HEK293T cells with and without *HRD1^–/–^* or *RNF5^–/–^*, with quantitation shown in **C** and Supplemental Figure 9, C and E (*n* = 3–9 per group). (**D**) Western blot analysis of HRD1 in WT or KI HEK293T cells with and without *HRD1^–/–^* using 2 different SEL1L antibodies, with quantitation shown in Supplemental Figure 9B (*n* = 3–6 per group). (**E** and **F**) Cycloheximide chase analysis of ERAD proteins in WT and KI HEK293T cells with and without *HRD1^–/–^*, with quantitation shown (**F**) (*n* = 3–6 per group). OS9 1 indicates isoform OS-9.1; OS9 2 indicates isoform OS-9.2. Both bands were quantitated together. (**G**) Reducing and nonreducing SDS-PAGE and Western blot analysis of HMW aggregates of SEL1L in KI HEK293T cells with and without *HRD1^–/–^* (representative of 2 repeats). Data are represented as means ± SEM. **P < 0.01; ***P < 0.001; ****P < 0.0001, 1-way ANOVA followed by Tukey’s post hoc test (**C**); 2-way ANOVA followed by Tukey’s multiple-comparisons test (**F**).

We next further explored mechanistically how the *SEL1L^C141Y^* variant causes the instability of the SEL1L-HRD1 ERAD complex. We first asked whether proteasomes are required in this process. Treatment with the proteasomal inhibitor MG132 elevated the protein levels of both SEL1L and HRD1 (Figure 7A and Supplemental Figure 9A), pointing to the involvement of the proteasomes in the reduction of the ERAD complex. Next, as previous studies have implicated HRD1 (41, 42) or RING finger protein 5 (RNF5/RMA1) E3 ligase (43) in HRD1 turnover, we asked which the E3 ligase is involved in the degradation of the SEL1L-HRD1 complex in the presence of the variants. We generated *HRD1^–/–^* or *RNF5^–/–^* HEK293T cells expressing (via KI) *SEL1L^C127Y^* and *SEL1L^C141Y^*. Strikingly, deletion of HRD1, but not RNF5, significantly rescued the protein levels and stability of SEL1L in *SEL1L^C127Y^* and *SEL1L^C141Y^* KI HEK293T cells (Figure 7, B, C, E, and F). This was consistent using 2 different SEL1L antibodies recognizing different regions of SEL1L protein (Figure 7D and Supplemental Figure 9B). The accumulation of SEL1L^C141Y^ protein in the absence of HRD1 protein led to the formation of HMW aggregates in HEK293T cells (Figure 7G). Similarly, OS9 and ERLEC1 proteins accumulated (Figure 7B and Supplemental Figure 9C) and formed HMW complexes (Supplemental Figure 9D) in *HRD1^–/–^;SEL1L^C127Y^* and *HRD1^–/–^;SEL1L^C141Y^* HEK293T cells. Additionally, both IRE1α and CD147 were also further increased upon the deletion of HRD1 compared with the parental *SEL1L^C127Y^* and *SEL1L^C141Y^* KI cells, pointing to residual HRD1 function in these KI cells (Figure 7B and Supplemental Figure 9E). In conclusion, *SEL1L^C141Y^* causes proteasome-mediated self-destruction of the SEL1L-HRD1 ERAD complex.

## Discussion

**Figure 8:**
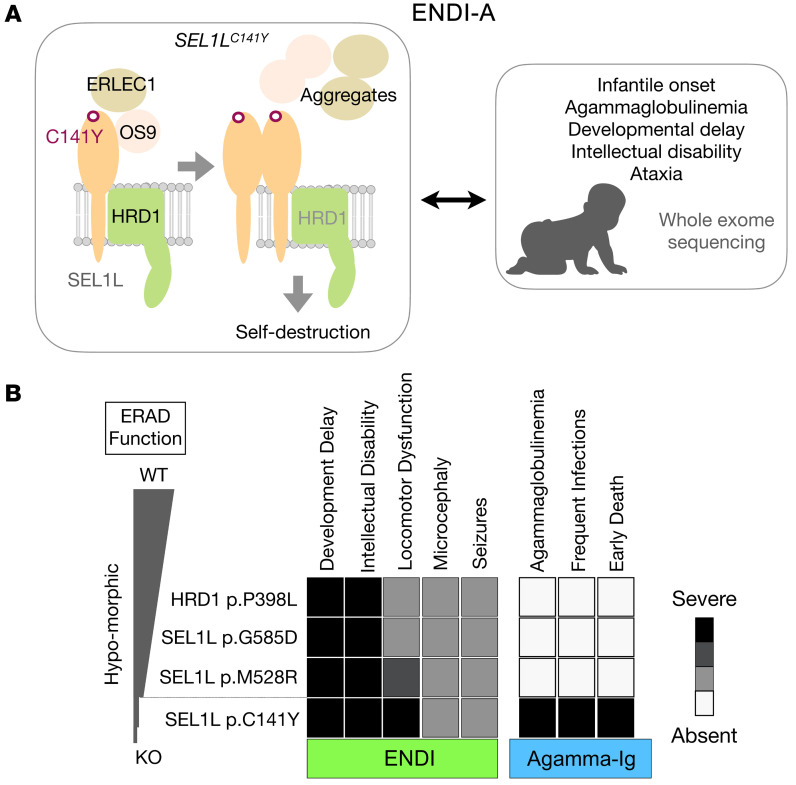
Our models for disease causality of *SEL1L^C141Y^* and an inverse correlation between ERAD function and disease severity in humans. (**A**) Human *SEL1L^C141Y^* variant causes a significant loss of SEL1L-HRD1 ERAD function due to aggregation and self-destruction, leading to ENDI-A. (**B**) In comparison with other variants, the *SEL1L^C141Y^* variant is much more severe in terms of ERAD dysfunction and disease severity.

Here we report a group of patients expressing a new biallelic *SEL1L^C141Y^* variant with clinical features of ENDI-A (Figure 8A). They resemble the ENDI patients described in the accompanying paper (32) in terms of neurodevelopmental disorders characterized by infantile-onset developmental delay, intellectual disability, microcephaly, hypotonia, and facial dysmorphisms, i.e., ENDI. However, unlike those ENDI patients, they exhibited severe B cell immunodeficiency, suffered frequent infections that required Ig replacement therapy, and died due to respiratory insufficiency. These differences likely reflect different degrees of ERAD dysfunction among these variants; while *SEL1L* (p.Gly585Asp, p.Met528Arg) and *HRD1* (p.Pro398Leu) variants are hypomorphic with moderate ERAD dysfunction, the *SEL1L* p.Cys141Tyr variant is much more severe, with a significant loss of the ERAD complex and much more severe ERAD dysfunction (Figure 8, A and B).

Agammaglobulinemias are congenital diseases characterized by a lack of functional B cells and antibodies (44). Here, we show that in humans, SEL1L-HRD1 ERAD dysfunction is likely associated with agammaglobulinemia. This finding is supported by previous reports that SEL1L-HRD1 ERAD plays a key role in B cell development in mice as a checkpoint to control the degradation and hence abundance of pre-BCR (11, 13). SEL1L-HRD1 deficiency in B cell lineage causes B cell developmental blockade at the large pre–B cell stage, leading to a significant reduction of mature B cells (11, 13). In comparison, the effect of *SEL1L* p.Cys141Tyr variant on T cells is more moderate with largely normal absolute number of T cells in the periphery. Nonetheless, the ratio of CD4^+^ to CD8^+^ T cells is altered, in line with a known role of SEL1L-HRD1 ERAD in αβ T cell development (15). Hence, given the severity of ERAD dysfunction, we believe that the *SEL1L^C141Y^* variant likely causes agammaglobulinemia by blocking B cell development in humans. Definitive evidence will come from further studies with mouse models carrying the variant, which will also be useful to delineate how T cell development and function are affected.

From combining evidence from genetics and in silico and in vitro analysis, *SEL1L^C141Y^* has been considered to be a pathogenic variant with a total score of 14 points based on ACMG criteria (34). At the same time, we also considered the possibility of a second biallelic variant causing immune deficiency phenotypes in ENDI-A. Other variants associated with neurodevelopmental disorders or hypogammaglobulinemia were examined independently with chromosome analysis, SNParray, and next-generation sequencing (NGS); however, these tests did not show any other genomic imbalances or other associated genetic variants. Genetically, the chance of 5 affected children showing similar symptoms of 2 different diseases with a genetic linkage of 2 different biallelic variants is extremely low. Therefore, we consider the *SEL1L^C141Y^* variant as the only possible causative variant in these patients. With that being said, we acknowledge that we cannot firmly establish disease causality without a KI mouse model carrying the variant.

SEL1L has 2 disulfide bonds, both of which are in the FNII domain. Here our data reveal the importance of disulfide bonds in the little-known FNII domain of SEL1L, while the FNII domain itself is dispensable in SEL1L-HRD1 function. The FNII domain probably formed during evolution via exon shuffling (45) and is also present in other proteins, such as coagulation factor XII (46) and the cation-independent mannose-6-phosphate/insulin-like growth factor-II receptor (IGF2R) (47). Disrupting either disulfide bond causes HRD1-mediated self-destruction of the complex, presumably due to the formation of aberrant disulfide bonds (Figure 8A). This effect of the disease variant is distinct from that of a simple loss of function of SEL1L, where there is no free cysteine. In the latter case, loss of SEL1L causes HRD1 self-degradation, while leading to the stabilization and accumulation of lectins such as OS9/ERLEC1 (Supplemental Figure 10).

While much more in disease pathogenesis associated with this variant awaits further investigation, the identification of this variant not only provides exciting opportunities for studying ERAD biology, but also further establishes the (patho-)physiological importance of SEL1L-HRD1 ERAD. Together with the findings reported in the accompanying paper (32), our data have uncovered an inverse correlation between SEL1L-HRD1 ERAD and disease severity in humans. It paves the foundation for future efforts to therapeutically target this important protein complex in the treatment of human diseases.

## Methods

### Human subjects.

The patient cases were gathered through the web-based tool GeneMatcher (48) (https://genematcher.org/statistics/). We present 5 developmentally delayed children with agammaglobulinemia. They were raised from birth in a big family of Roma population living in an isolated region in the southern part of Slovakia. Patients 1 and 2 (IV-1 and IV-2) were born in 2006 and 2014, respectively, from a pair of consanguineous parents in the family.

Patient 1 (IV-1) presented hypotonia, developmental delay, frequent vomiting after eating, facial dysmorphisms, and agammaglobulinemia since her birth. At 15 months, she developed a 30% weight deficit, representing dystrophy of the third degree (marasmus). The physical examination revealed at the age of 3 years of life, a short stature (–3.28 SD), underweight (–5.81 SD), microcephaly (–1.89 SD), and severe developmental delay (Supplemental Table 2). She was treated because of acute renal failure and pulmonary hypertension. She died due to multiorgan failure at the age of 2.9 years. Patient 2 (IV-2) presented axial hypotonia and facial dysmorphism since her birth. She got bronchopneumonia and died suddenly 2 months after birth. Her Ig levels were not tested.

Patients 3–5 (IV-3, IV-5, and IV-6) were born in 2009, 2014, and 2016, respectively, from another pair of consanguineous parents in the family. All 3 patients presented with hypotonia, developmental delay, hypotrophy, facial dysmorphism, repeated vomiting after eating, and agammaglobulinemia since birth. Patient 3 (IV-3) was born with a ventricular septal defect. He was delivered at 37 weeks of gestation, birth weight of 2700 g (percentile = 25th) and length of 48 cm (percentile = 50th) with normocephaly. Because of the episode of unspecified epilepsy, a brain MRI was done, but showed normal myelinization. He was treated by valproate therapy. The genetic examination for cystic fibrosis and the hereditary agammaglobulinemia-*BTK* gene was negative. The child lived later in an orphanage, and his last physical examination showed at the age of 2.5 years short stature (–2.62 SD) and that he was underweight (–4.21 SD) (Supplemental Table 2). He was lying in bed, exhausted from severe sepsis, and died from respiratory failure. Patient 4 (IV-5) did brain MRI at the age of 10 months, showing unspecific leukoencephalopathy frontal and occipital bilaterally, but she had no seizures. At the age of 6 years, she could turn, sit with support, and tried to stand with support. She didn’t speak. At the age of 4.5 years, her physical examination showed short stature (–3.12 SD), underweight (–3.03 SD), and microcephaly with 43 cm (–2.83 SD). Eye examination showed palpebral ptosis with bilateral partial papillary atrophy. Laboratory examination showed sideropenic anemia, increased folate level, and hypovitaminosis D. She was treated every 4 weeks with intravenous Ig, silymarin, hepatoprotective essential phospholipids, and pyridoxine, and during infection, with antibiotics. Two months before she died, she became sick with COVID-19 with a prolonged course compared with that of other children with hereditary agammaglobulinemia.

Patient 5 (IV-6) was born at 39 weeks gestation with neurotrophic data (3025 g/50 cm) with microcephaly (head circumference: 31 cm, >3 pc). At the age of 2.5 years, physical examination revealed short stature (–2.25 SD), that he was underweight (–2.64 SD), microcephaly (–2.64 SD), hypotonia, and severe developmental delay (Supplemental Table 2). Brain MRI was done and revealed the leukoencephalopathy frontal, parietal, and occipital bilaterally and later, at the age of 4.5 years, discrete supratentorial cortical atrophy. He did not develop seizures. The eye examination showed bilateral palpebral ptosis and bilateral papillary excavation. He had a micropenis, central hypothyroidism, hypoplastic thymus, hepatopathy, and dystrophic nails. He was intensively treated with antibiotics and oxygen and every 4 weeks with antibody supplementation therapy. At 5.1 years, he died due to severe pneumonia and respiratory failure.

Patient IV-4 was a girl, born with severe hypotonia, alobar holoprosencephaly, and multiple malformations. The patient presented cheilognathopalatoschisis, 1 nostril with missing part of nasal wings, “frog” eyes, almost closed vision field, low-set deformed ears, microcephaly, short neck, pterygium colli, narrower chest, transverse groove on the left hand, 4 fingers with claw-like position on the right hand, and feet with malformed fingers with polydactyly and syndactyly of fourth and fifth fingers. The anus was malformed; multiple contractures on all joints were seen. She presented congenital heart defects with atrial ventricular septum defect and patent ductus arteriosus. The patient died at 9 days because of cardiorespiratory failure. The patient had no infection or problem with immunity as in the other siblings with agammaglobulinemia.

### CRISPR/Cas9-based KO and KI HEK293T cells.

HEK293T cells, obtained from ATCC, were cultured at 37°C with 5% CO_2_ in DMEM with 10% fetal bovine serum (Fisher Scientific). To generate SEL1L-, HRD1- and RNF5-deficient HEK293T cells, sgRNA oligonucleotides designed for human *SEL1L* (5′-GGCTGAACAGGGCTATGAAG-3′), human *HRD1* (5′-GGACAAAGGCCTGGATGTAC-3′), or human *RNF5* (5′-CACCTGTACCCCGGCGGAA-3′) were inserted into lentiCRISPR, version 2 (Addgene 52961). Cells grown in 10 cm petri dishes were transfected with indicated plasmids using 5 μl 1 mg/ml polyethylenimine (PEI) (MilliporeSigma) per 1 μg of plasmids for HEK293T cells. The cells were cultured 24 hours after transfection in medium containing 2 μg/ml puromycin for 24 hours and then in normal growth medium.

*SEL1L^C141Y^* KI HEK293T cells were generated as described in the accompanying paper by Wang et al. (32). ΔFNII KI HEK293T cells were generated using the CRISPR/Cas9 approach. We designed 2 gRNAs (gRNA1 and gRNA3) flanking the *SEL1L* exon 4, which encodes the SEL1L FNII domain, and an additional gRNA (gRNA2) in close proximity to gRNA1 to enhance editing efficiency. These gRNAs were synthesized by Integrated DNA Technologies (IDT). These gRNAs and Cas9 protein were introduced into the cells via electroporation, followed by culturing and single-cell isolation with the desired genomic modification. *SEL1L^C127Y^* KI HEK293T cells were generated using the CRISPR/Cas9 cytosine base editing (CBE) system (49). Oligos with the gRNA sequence were annealed at 95°C for 5 minutes and cooled to room temperature for 30 minutes. The duplex was inserted to BbsI-treated (NEB) and gel-purified pYZ122-pSMART HCKan-sgRNA-Sp-BbsI plasmid, a gift from the Yan Zhang Laboratory (University of Michigan Medical School). The ligated product was transformed and amplified in DH5-α *E*. *coli* cells, and the plasmid sequence was confirmed by Sanger sequencing (rurofins). To transfect HEK293T cells, 125 ng of the plasmid containing the gRNA sequence and 375 ng of pCAG-CBE4max-SpG-P2A-EGFP (Addgene, 139998) were introduced to the cells with a confluency of 80% via Lipofectamine 3000 (Thermo Fisher) in 1 well of a 24-well plate.

The CRISPR-processed cells were cultured at 37°C with 5% CO_2_. After 3 days of incubation, the genomic DNA of the cell culture was extracted with 50 mM NaOH. DNA fragments covering the target sites were amplified by PCR, using HotStart Taq 2× PCR Master (ABclonal), and analyzed by Sanger sequencing (eurofins) to estimate the percentages of mutant allele in the cell pool. In parallel, the cell culture was diluted into 8 cells per mL and cultured in 96-well plates (100 μL per well) for single-cell isolation. After 10 days, 100 single-cell colonies were transferred into 24-well plates. The *SEL1L^C141^* or *SEL1L^C127^* region of each colony was amplified using a 20 μL PCR reaction and sequenced. Cell colonies with homozygous *SEL1L^C141Y^* or *SEL1L^C127^* alleles were selected and transferred into 6-well plates for further experiments. For the ΔFNII KI cell line, total RNA was extracted using TRI Reagent and BCP Phase Separation Reagent (Molecular Research Center, TR 118), followed by cDNA library generation using the High Capacity cDNA Reverse Transcription Kit (Thermo Fisher). The region encoding the FNII domain was amplified using a 20 μL PCR reaction and sequenced. Sequences were as follows: *SEL1L^C141Y^* crRNA (guide sequence): guide 1: 5′-ATGAATGTACATCAGATGGG-3′, guide 2: 5′-ATTCATCATACTCCTTATCT-3′; ΔFNII crRNA (guide sequence): guide 1: 5′-GGTAACTTCCGTGTCGTGTA-3′, guide 2: 5′-AACTTCCGTGTCGTGTACCC-3′, guide 3: 5′-ACTACAAAGCAGATGAAAAG-3′; HDR donor oligo(mutation sites are underlined): *SEL1L^C141Y^*: 5′-CACTTCCCTTTTCTTTTCCTAGATAAGGAGTATGATGAATATACATCAGATGGGAGGGAAGATGGCAGACTGTGGTGTGCTACAACCT-3′, *SEL1L^C127Y^* gRNA oligos (gRNA sequence is underlined): F: 5′-CACCGAGTGGCAGGGCTCCCCATG-3′, R: 5′-AAACCATGGGGAGCCCTGCCACTc-3; amplification PCR primers: *SEL1L^C127Y^* and *SEL1L^C141Y^*: F: 5′-TCAGCTAGCCATGCTCACTAAA-3′, R: 5′-TGACTTGAGTGACAGCCTGAAA-3′; ΔFNII: F: 5′-CTGCAGGCAGAGTAGTTGCT-3′, R: 5′-TGCATCTGCCGTCTCTTAGC-3′.

### Plasmids.

The following plasmids were used in the study (h denotes human genes; m denotes mouse genes): *mSel1L* cDNA was cloned from mouse liver cDNA and inserted into the pcDNA3 to generate pcDNA3-mSEL1L(WT)-FLAG. pcDNA3-h-proAVP(G57S)-HA were described previously (24). *SEL1L^C141Y^* mutations in this study were generated using site-directed mutagenesis with pcDNA3-mSEL1L(WT)-FLAG as the template. All plasmids were validated by DNA-Seq. The cloning primers were as follows: mSEL1L-FLAG-F: 5′-CGCGGATCCACCATGCAGGTCCGCGTCAGGCTGTCG-3′; R: 5′-CGCTCTAGACTATTTATCATCATCATCTTTATAATCTCCGCCCTGTGGTGGCTGCTGCTCTGG-3′. C141Y-FLAG-F: 5′-GTATGATGAGTACACCTCAGACG-3′; R: 5′-CGTCTGAGGTGTACTCATCATAC-3′.

### Western blot and antibodies.

Cells were harvested and snap-frozen in liquid nitrogen. The proteins were extracted by sonication in NP-40 lysis buffer (50 mM Tris-HCl at pH7.5, 150 mM NaCl, 1% NP-40, 1 mM EDTA) with protease inhibitor (MilliporeSigma), DTT (MilliporeSigma, 1 mM), and phosphatase inhibitor cocktail (MilliporeSigma). Lysates were incubated on ice for 30 minutes and centrifuged at 16,000*g* for 10 minutes. Supernatants were collected and analyzed for protein concentration using Bio-Rad Protein Assay Dye (Bio-Rad). From 10 to 30 μg of protein was denatured at 95°C for 5 minutes in 5× SDS sample buffer (250 mM Tris-HCl pH 6.8, 10% sodium dodecyl sulfate, 0.05% bromophenol blue, 50% glycerol, and 1.44 M β-mercaptoethanol). Protein was separated using SDS-PAGE, followed by electrophoretic transfer to PVDF (Fisher Scientific) membrane. The blots were incubated in 2% BSA/TBST with the following primary antibodies overnight at 4°C: anti-HSP90 (Santa Cruz Biotechnology Inc., sc-13119, 1:5,000), anti-SEL1L (homemade, against SEL1L, 23–205 aa, ref. 50, 1:10,000), anti-SEL1L (Abcam, ab78298, against SEL1L, 330–400 aa, 1:1000), anti-HRD1 (Proteintech, 13473-1, 1:2,000), anti-OS9 (Abcam, ab109510, 1:5,000), anti-ERLEC1 (Abcam, ab181166, 1:5,000), anti-CD147 (Proteintech, 11989-1, 1:3,000), anti-IRE1α (Cell Signaling Technology, 3294, 1:2,000), anti-UBE2J1 (Santa Cruz Biotechnology Inc., sc-377002, 1:3,000), anti-ubiquitin (Santa Cruz Biotechnology Inc., P4D1, 1:1000), anti-LC3 (Cell Signaling Technology, 2775), anti-RNF5 (Bethyl, A303-594A, 1:2000), anti-FLAG (MilliporeSigma, F1804, 1:1,000), anti-HA (MilliporeSigma, H3663, 1:5,000), anti-PERK (Cell Signaling Technology, 3192, 1:5000), anti-–p-PERK (Cell Signaling Technology, 3179, 1:1,000), anti-eIF2α (Cell Signaling Technology, 9722, 1:5000), anti-p-eIF2α (Cell Signaling Technology, 9721, 1:1000), anti-BiP (Abcam, ab21685, 1:5,000), and anti-PDI (Enzo, ADI-SPA-890, 1:5,000). Membranes were washed with TBST and incubated with HRP-conjugated secondary antibodies (Bio-Rad, 1:10,000) at room temperature for 1 hour for ECL Chemiluminescence Detection System (Bio-Rad) development. Band intensity was determined using Image Lab (Bio-Rad) software, version 6.1.

For additional information, see Supplemental Methods.

### Statistics.

Statistics tests were performed using GraphPad Prism, version 8.0 (GraphPad Software). Unless indicated otherwise, values are represented as means ± SEM. All experiments were repeated at least 2 to 3 times and/or performed with multiple independent biological samples from which representative data are shown. All data sets passed normality and equal variance tests. Statistical differences between the groups were compared using unpaired 2-tailed Student’s *t* test for 2 groups or 1-way ANOVA or 2-way ANOVA for multiple groups. *P* < 0.05 was considered statistically significant.

### Study approval.

Study protocols and protocols for written, informed consent were approved by the Johannes Kepler University Ethics Committee (JKU-EC, approval no. 1253/2021), the Institutional Review Boards of the University of Michigan Medical School (IRBMED, HUM00227482), and the Institutional Review Board for Health Sciences Research (IRB-HSR, University of Virginia, HSR230351). Patients and parents provided written, informed consent prior to participation in the study. Written, informed consent was received for use of the photographs.

### Data availability.

Materials and reagents used are either commercially available or available upon request. All materials used for the manuscript are included in Methods. Values for all data points in graphs are reported in the Supporting Data Values file.

## Author contributions

DW, HMW, ALP, SW, KS,GH, AS, LD, and JAM obtained clinical, molecular, and biochemical data. LLL, HHW, ZJL, ZW, XW, and TTY designed and performed biochemical experiments. KK and RGF assisted with some experiments and analysis. PC acquired clinical and immune profile data. DW, RGF, JAM, and LQ directed the study. DW, PC, HHW, LLL, and LQ wrote the manuscript. All authors commented on and approved the manuscript.

## Supplementary Material

Supplemental data

Supporting data values

## Figures and Tables

**Table 2:**
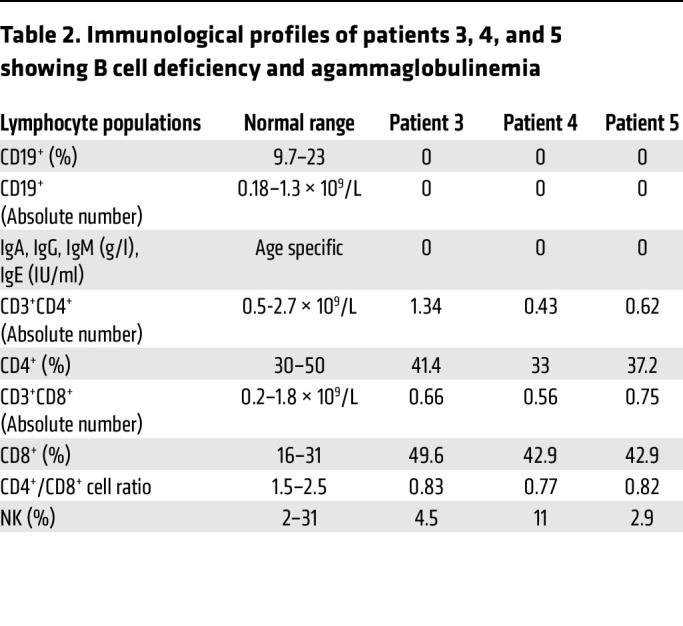
Immunological profiles of patients 3, 4, and 5 showing B cell deficiency and agammaglobulinemia

**Table 1:**
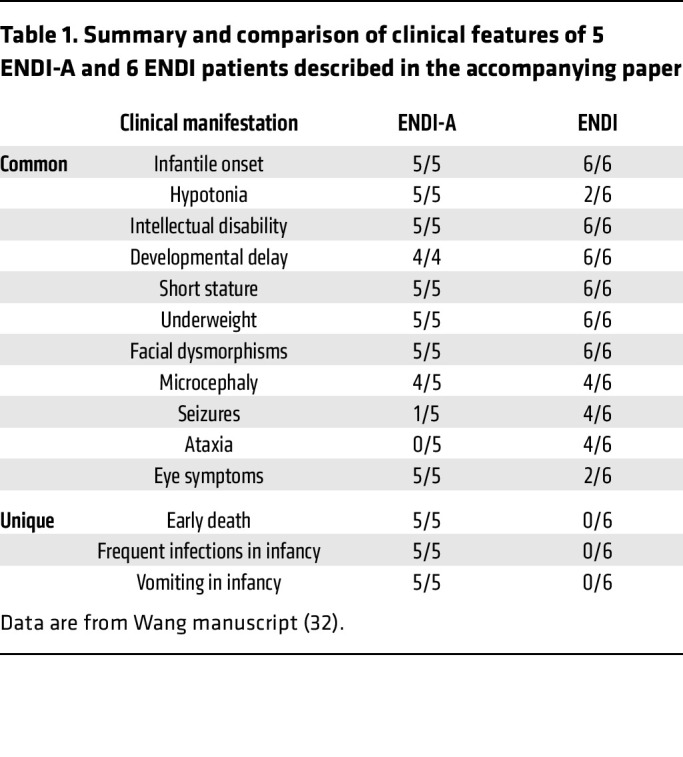
Summary and comparison of clinical features of 5 ENDI-A and 6 ENDI patients described in the accompanying paper

## Version 1 11/09/2023

In-Press Preview

## Version 2 01/16/2024

Electronic publication
